# Hybrid surgical approach for chronic pancreatitis: pyloric-ring-resection pancreaticoduodenectomy with lateral pancreatojejunostomy

**DOI:** 10.1093/jscr/rjag180

**Published:** 2026-03-22

**Authors:** Omar Barakat, Jared R Mortus, Claire F Ozaki

**Affiliations:** Division of Surgical Oncology and Hepatobiliary and Pancreas Surgery, Baylor College of Medicine, 7200 Cambridge St, 7th Floor, Houston, TX 77030, United States; Department of Surgery, Baylor College of Medicine, One Baylor Plaza, Houston, TX 77030, United States; Department of Surgery, Baylor College of Medicine, One Baylor Plaza, Houston, TX 77030, United States

**Keywords:** chronic pancreatitis, pancreaticoduodenectomy, lateral pancreatojejunostomy, Puestow procedure, Whipple, bile duct stricture

## Abstract

Pancreaticoduodenectomy (PD) and lateral pancreatojejunostomy are established surgical techniques for treating chronic pancreatitis (CP), but their combination has not been studied. We performed PD combined with lateral pancreatojejunostomy in 15 patients with morphological changes consistent with CP. Preoperative imaging revealed features of CP confined to the head of the pancreas (HOP) and main pancreatic duct (MPD) dilation with strictures and stones in all but one patient. Surgical procedures were completed successfully in all the patients. One patient (6.6%) developed major complications that required reoperation. There was no 90-day mortality. At follow-up (median, 66 months), pain, quality of life, and exocrine function had improved, whereas endocrine function worsened in 4 of the 7 non-insulin-dependent diabetic patients, requiring insulin therapy. This hybrid approach provided durable pain relief, improved quality of life, and stabilized exocrine function. Endocrine deterioration in some cases highlights the challenges of managing pre-existing pancreatic insufficiency.

## Introduction

Chronic pancreatitis (CP) is a debilitating inflammatory disease of the pancreas marked by progressive fibrosis and permanent loss of both endocrine and exocrine functions, resulting in diabetes and malabsorption. Moreover, approximately one-third of patients develop complications due to the extension of the inflammatory process into adjacent organs and vascular structures, leading to portal vein thrombosis and strictures of the bile duct and duodenum [1]. Chronic abdominal pain is the most common and debilitating symptom, significantly affecting patient quality of life.

Surgical procedures for CP generally include pancreatic resection, pancreatic duct drainage, or a combination of both. Resection procedures include (i) pancreaticoduodenectomy (PD) or Whipple procedure, typically reserved for patients with CP affecting the head of the pancreas (HOP), especially cases with bile duct or duodenal obstruction or suspected malignancy [2–5]; (ii) distal pancreatectomy, commonly performed for CP affecting the body and tail of the pancreas [6–8]; and (iii) total pancreatectomy with or without islet cell transplant, used to treat severe CP involving the entire pancreas [9–11].

For patients with CP with involvement of the peripancreatic vasculature, drainage approaches involve lateral pancreatojejunostomy (PJ; modified Puestow or Partington–Rochelle procedure), the preferred method for patients with a dilated main pancreatic duct (MPD) > 5 mm and no mass in the HOP. This procedure offers low morbidity and excellent long-term pain relief [12–14]. For patients with localized inflammatory changes in the HOP, duodenum-preserving pancreatic head resection (DPPHR; Frey, Berne, and Beger procedures), in which the HOP is either partially removed or cored out while preserving the duodenum, is used, followed by drainage of the MPD [15, 16].

In this case series of 15 patients with CP confined to the HOP and multiple strictures and stones involving the MPD along the body and tail of the pancreas but with no involvement of the peripancreatic vasculature, we describe a novel hybrid surgical approach that combines pyloric-ring-resection PD with lateral PJ. Unlike DPPHR, this approach can be used in patients with no involvement of the peripancreatic vasculature. To our knowledge, such a hybrid procedure has not been reported or evaluated in terms of feasibility, short-term and long-term pain relief, endocrine and exocrine functions, and postoperative complications.

## Case series

### Patients and preoperative assessment

Between 2010 and 2023, 15 patients exhibiting morphological changes consistent with CP (such as calcifications and intraductal stones) confined to the HOP and a dilated MPD underwent pyloric-ring-resection PD (Whipple procedure) and side-to-side drainage of the MPD. Preoperatively, all the patients were routinely evaluated with pancreatic protocol contrast-enhanced computed tomography scans and magnetic resonance cholangiopancreatography. The preoperative data collected included age, sex, ethnicity, clinical symptoms of CP, symptom duration, narcotic addiction and duration, Charlson Comorbidity Index, number of preoperative endoscopic procedures, and quality of life as measured using EuroQol 5 Dimensions with 5 levels (EQ-5D-5L) questionnaires completed by patients with higher scores reflecting poorer quality of life.

The morphology of the diseased pancreas was recorded, including the maximal diameter of the MPD and the presence of ductal stones, strictures, and calcifications. Extrapancreatic complications, such as bile duct stricture, duodenal stricture, and pseudocyst formation, also were noted.

The perioperative and postoperative data included operative time, estimated blood loss, type of reconstruction, surgery-related complications, length of hospital stay, reoperation within 90 days, readmission rates, and mortality at 90 days and 12 months. Additionally, we assessed changes in pancreatic endocrine and exocrine functions, postoperative pain control, opioid use, and quality of life.

Postoperative complications were graded by severity according to the Dindo–Clavien classification system, adapted for pancreatic surgery [17]. Postoperative pancreatic fistula (POPF) is now redefined as any clinically relevant POPF (grade B/C) that has a drain output of any measurable volume and an amylase level > 3 times the upper limit of the institutional normal serum amylase value. Delayed gastric emptying was defined according to the consensus categorization (A, B, or C) proposed by the International Study Group of Pancreatic Surgery [18].

### Surgical technique

All the patients underwent PD as previously described [19]. After resection, the MPD was inspected using an intraoperative pancreatoscope introduced through the opening of the MPD at the cut surface of the pancreas, to confirm the presence of strictures and intraductal stones. The MPD was then incised longitudinally to release the strictures and facilitate stone extraction.

Next, the proximal end of the first loop of jejunum was brought anterior to the transverse colon to complete a side-to-side gastrojejunostomy to the posterior wall of the stomach, using a gastrointestinal tri-stapling device (Covidien, Dublin, IE). The jejunum was then divided 45 cm distal to the gastrojejunostomy, and the distal limb was brought separately through the transverse mesocolon for the reconstruction of a side-to-side PJ and an end-to-side hepaticojejunostomy (HJ).

**Figure 1:**
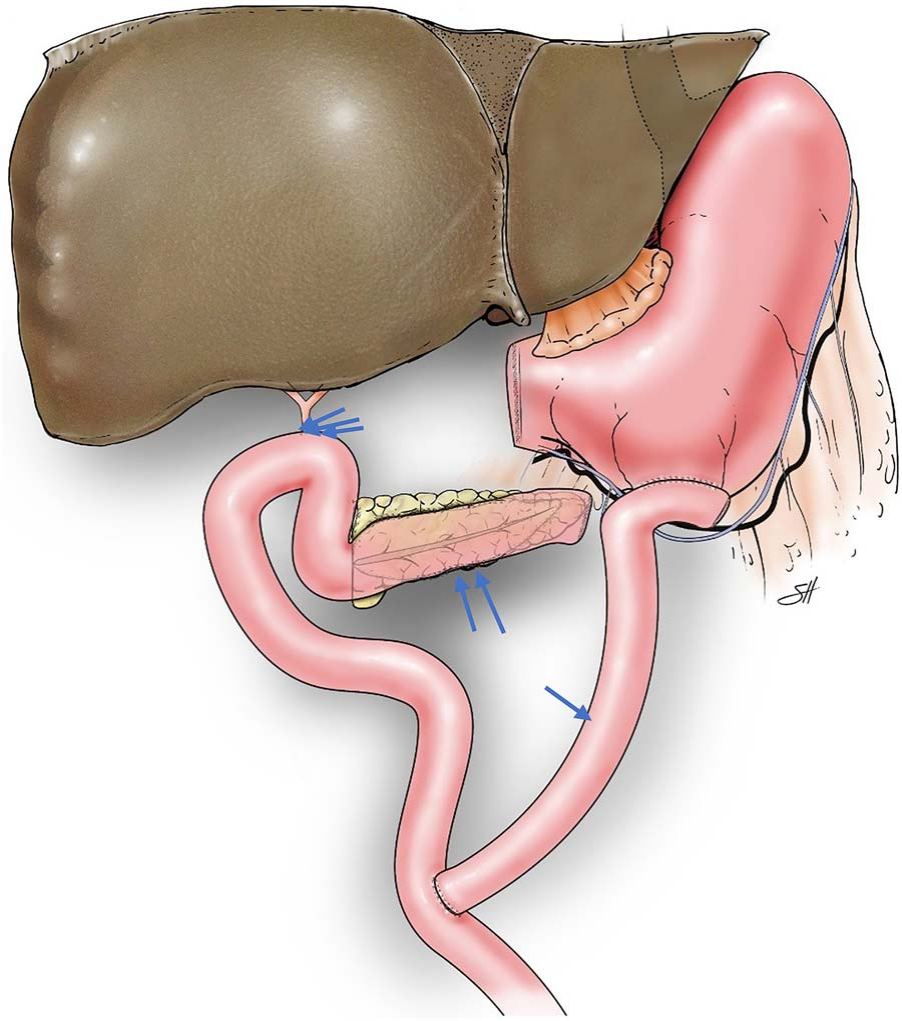
Illustration depicting reconstruction of a Roux-en-Y 45-cm proximal gastrojejunal anastomosis (arrow), distal side-to-side pancreatojejunostomy (double arrows), and 50-cm hepaticojejunostomy (triple arrows) after pyloric-ring–resection pancreaticoduodenectomy. ©Baylor College of Medicine.

**Figure 2:**
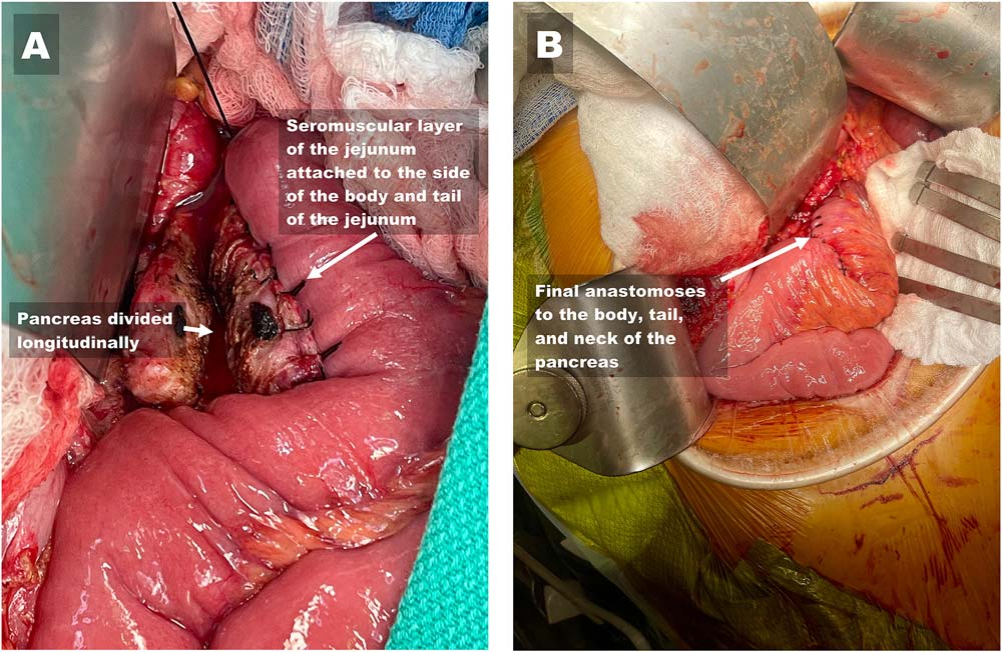
Reconstruction of the side-to-side pancreatojejunostomy to the body, tail, and neck of the pancreas. (A) Image showing the divided main pancreatic duct (left arrow) after completion of the first layer of the side-to-side pancreatojejunostomy (right arrow) and the neck of the pancreas. (B) Image showing the completed two-layered anastomosis.

The PJ was performed in two layers using 3-0 silk and 4-0 Prolene sutures to connect the jejunal loop to the entire length of the incised MPD. Upon reaching the cut end of the pancreas, the jejunal loop was rotated 90 degrees to continue the anastomosis over the cut surface of the neck (Figs 1 and 2). In 10 patients, the HJ was constructed using the same jejunal loop, positioned 15 cm distal to the PJ. In 4 patients, the jejunum was divided ~15–20 cm distal to the PJ, and the distal limb was brought up to complete the end-to-side HJ in one layer using 5-0 polydioxanone sutures in an interrupted fashion. The first jejunal–jejunal anastomosis was constructed ~20 cm from the HJ, and the second jejunal–jejunal anastomosis was constructed ~50 cm from the HJ. All the jejunal anastomoses were completed using the Covidien tri-stapling device. In one patient, a gastric interposition graft between the proximal hepatic duct and the PJ jejunal loop was used to construct the HJ, as previously described [20].

A feeding jejunostomy was not placed. In all cases, two Jackson-Pratt silicone drains were introduced through separate stab incisions and were positioned anterior and posterior to the PJ and HJ, respectively.

### Perioperative management

After the procedure, patients were admitted to the surgical floor. The nasogastric tube was removed when the volume of gastric content was <500 ml/day. All the patients were placed on proton pump inhibitors. Serum amylase and drain-fluid amylase levels were measured on postoperative days 1 and 3. Drains were removed if no evidence of pancreatic or bile leakage was present. Serum glucose level was maintained within normal limits by using an insulin sliding scale.

When patients were clinically well and tolerated a postgastrectomy soft diet, they were discharged from the hospital.

### Outcomes

Patient characteristics and preoperative data are shown in Table 1. The cohort had a median age of 56 (range, 40–73) years and was predominately female (*n* = 11) and White (*n* = 11). Chronic pancreatitis was attributed to biliary stones in 6 patients and to alcohol addiction in 8; 1 patient had hereditary pancreatitis. The primary symptoms observed were chronic pain and steatorrhea, affecting 14 patients, with a median fecal elastase value of 169 μg/g (range, 120–200 μg/g). Among 8 patients with diabetes mellitus, 7 were non-insulin-dependent preoperatively and 1 was insulin-dependent.

**Table 1:** Preoperative variables for 15 patients who underwent a combined Puestow and Whipple procedure

Case	1	2	3	4	5	6	7	8	9	10	11	12	13	14	15
Age (years)	73	51	56	72	54	40	60	69	73	56	56	72	54	40	61
Sex	F	F	F	M	F	F	F	M	F	F	M	F	F	F	M
Race	W	B	W	B	W	W	W	W	W	B	B	W	W	W	W
Etiology	Biliary	Biliary	Alcohol	Alcohol	Biliary	Alcohol	Alcohol	Hereditary	Biliary	Biliary	Alcohol	Alcohol	Alcohol	Alcohol	Alcohol
Symptoms	ChP/OJ	ChP	ChP	OJ	ChP/OJ	ChP	ChP	ChP	ChP/OJ	ChP	ChP	ChP/OJ	ChP	ChP	ChP
Steatorrhea	No	Yes	Yes	Yes	Yes	Yes	Yes	Yes	Yes	Yes	Yes	Yes	Yes	Yes	Yes
Pain score	8/10	9/10	9/10	0/10	4/10	10/10	8/10	10/10	7/10	9/10	10/10	6/10	9/10	8/10	9/10
NIDDM	Yes	No	No	Yes	Yes	Yes	No	No	Yes	No	Yes	No	Yes	No	No
IDDM	No	No	No	No	No	No	No	Yes	No	No	No	No	No	No	No
Symptom duration (years)	3	2	4	15	4	8	2	3	2	2	10	6	8	2	4
CCI	3	4	3	5	3	2	2	6	3	4	2	5	2	2	5
BMI	33	40	18	20	23	20	31	24	36	20	20	31	24	40	18
Narcotic addiction	Yes	Yes	Yes	No	Yes	Yes	Yes	Yes	Yes	Yes	Yes	Yes	Yes	Yes	Yes
Duration of narcotic addiction (years)	2	2	4	0	4	1	1	2	1	1	2	0.5	1	1	1
Endoscopic procedure	Yes	Yes	Yes	No	Yes	Yes	No	Yes	Yes	Yes	Yes	No	Yes	Yes	Yes
Preoperative surgery	Yes	Yes	Yes	No	No	No	No	Yes	No	Yes	No	No	No	No	No
Quality of life	11	12	15	7	14	13	16	12	11	12	15	14	16	16	18

Abbreviations: B, Black non-Hispanic; BMI, body mass index; CCI, Charlson Comorbidity Index; ChP, chronic pain; F, female; IDDM, insulin-dependent diabetes mellitus; M, male; NIDDM, non-insulin dependent diabetes mellitus; OJ, obstructive jaundice; W, White non-Hispanic.

The median symptom duration was 4 years (range, 2–15 years). The median Charlson Comorbidity Index was 3 (range 2–6), and the median body mass index was 24 (range, 18–40). Notably, 14 patients had a history of preoperative narcotic addiction for a median of 1 year (range 0.5–4.0 years). Twelve patients had previously undergone endoscopic procedures to address strictures in the pancreatic and bile ducts, and 5 patients had undergone previous abdominal surgeries, including cholecystectomy (5 patients), choledochojejunostomy (1 patient), and cystgastrostomy (1 patient). The median quality of life score was 14 (range, 7–18).

Preoperative pancreatic morphology is detailed in Table 2. Imaging revealed a median MPD diameter of 7 mm (range, 5–12 mm). Twelve patients exhibited calcifications, mostly within the HOP. Additionally, 14 patients presented with strictures or stones in the MPD, with 8 of these patients also having concomitant biliary strictures. One patient displayed signs of duodenal stricture. Importantly, no patient had mass in the HOP or portal vein thrombosis. However, one patient had a pancreatic cyst, and one had a perisplenic abscess.

**Table 2:** Preoperative pancreatic morphology in 15 patients.

Case	1	2	3	4	5	6	7	8	9	10	11	12	13	14	15
Diameter of main pancreatic duct (mm)	5	5	7	12.0	6	8	6.7	8.4	6.1	7.8	6.4	7	8	6	7.8
Calcifications	Yes	No	Yes	Yes	No	Yes	No	Yes	Yes	Yes	Yes	Yes	Yes	Yes	Yes
Main pancreatic duct strictures	Yes	Yes	Yes	Yes	Yes	Yes	Yes	No	Yes	Yes	Yes	Yes	Yes	Yes	Yes
Stones	Yes	No	Yes	Yes	Yes	Yes	Yes	Yes	Yes	Yes	Yes	Yes	Yes	Yes	Yes
Biliary strictures	No	Yes	Yes	Yes	No	No	No	No	Yes	Yes	Yes	No	No	Yes	Yes
Duodenal stricture	No	No	Yes	No	No	No	No	No	No	No	No	Np	No	No	No
Mass in the head of the pancreas	No	No	No	No	No	No	No	No	No	No	No	No	No	No	No
Portal vein thrombosis	No	No	No	No	No	No	No	No	No	No	No	No	No	No	No
Extrapancreatic complications	No	No	Yes	No	No	Yes	No	No	No	No	No	No	No	No	No

Intraoperative and postoperative outcomes are shown in Table 3. Intraoperatively, the median surgical procedure time was 390 minutes (range, 250–570 minutes), with an estimated median blood loss of 137.5 ml (range, 100–1100 ml). In 10 patients, biliary reconstruction was successfully completed using the same loop of the PJ. However, four patients required an additional Roux-en-Y to construct the HJ due to a kink at the biliary anastomosis when using the same jejunal loop, and in one patient a gastric interposition graft was created to connect the proximal hepatic duct to the PJ jejunal loop. In 10 patients, the nasogastric tube was removed at the end of the procedure.

**Table 3:** Intraoperative and postoperative results in 15 patients.

Case	1	2	3	4	5	6	7	8	9	10	11	12	13	14	15
Length of procedure (minutes)	375	570	480	250	441	570	390	280	570	480	264	555	365	275	360
Estimated blood loss (ml)	300	1100	100	150	100	120	100	130	240	250	145	150	100	120	160
Days to start of liquid diet	2	3	2	1	1	2	1	1	3	2	1	1	2	1	1
Complication grade	No	II	No	No	No	No	IV	No	I	No	No	No	No	I	No
Postoperative pancreatic fistula	No	Yes	No	No	No	No	No	No	No	No	No	No	No	No	No
Delayed gastric emptying	No	No	No	No	No	No	No	No	No	No	No	No	No	No	No
Length of stay	7	15	4	9	8	8	4	3	13	4	9	8	4	3	4
Readmission	No	Yes	Yes	No	No	No	Yes	No	Yes	No	No	No	No	No	No
Follow-up (months)	180	120	12	60	47	12	10	89	156	132	16	66	96	108	60
Pain score	3	2	3	0	1	0	1	1	2	4	2	1	3	1	3
Exocrine insufficiency (steatorrhea)															
Improved	Yes	Yes			Yes	Yes	Yes	Yes		Yes		Yes	Yes		Yes
Stable			Yes	Yes					Yes		Yes			Yes	
Endocrine insufficiency (*n* = 8)															
Worsened	Yes			Yes							Yes		Yes		
Stable					Yes	Yes		Yes	Yes						
Quality of life	7	8	10	7	9	8	11	7	6	8	10	9	8	10	11
Narcotics	No	No	Yes	No	No	No	Yes	No	No	No	No	No	No	No	No

Postoperatively, patients resumed a clear liquid diet between days 1 and 3 and transitioned to soft foods between days 3 and 7. Three patients developed grade I/II complications, while one patient suffered a grade IV complication that required reoperation and admission to the ICU. One patient (6.6%) developed grade B POPF. None of the patients experienced delayed gastric emptying. The median length of hospital stay was 5 days (range, 3–15), with four patients (26.7%) requiring readmission within 90 days. None of the patients died within 12 months of surgery.

Long-term outcomes were promising, with a median follow-up period of 66 months (range, 10–180 months). Median pain scores improved significantly, decreasing from 9 to 2 (range, 0–4) on a 0–10 scale. Steatorrhea improved in 10 patients and remained stable in 5. These results translated to a 6-point improvement in the median quality-of-life score. However, 4 of the 7 patients with preoperative non-insulin-dependent diabetes mellitus experienced worsening conditions and became insulin-dependent.

## Discussion

The optimal surgical approach for patients with CP should aim to achieve effective pain relief, reduce or eliminate opioid use, stabilize remaining exocrine and endocrine function, improve quality of life, and address certain complications involving adjacent structures, such as duodenal or bile duct strictures. The choice of surgical procedure is typically guided by the specific morphological changes in the pancreas, involvement of adjacent structures (such as duodenal and bile duct strictures, portal vein thrombosis), and presence of pancreatic pseudocysts.

Over a 13-year period, we identified 15 patients with CP confined to the HOP with dilated MPD > 5 mm; 14 of these had multiple intraductal stones and strictures. Eight patients had bile duct stricture with intrahepatic and extrahepatic biliary dilation. Preoperative cross-sectional images did not reveal involvement of the portal vein or superior mesenteric vein. For 14 patients, chronic pain was the main symptom.

Short-term outcomes were similar to our previously reported PD results, in terms of the length of the operating time, intraoperative blood loss, and overall postoperative complications, including POPF and delayed gastric emptying [19]. One patient (6.6%) developed grade IV complications related to bile leak, intra-abdominal sepsis, and hemorrhage that required reoperation.

All the patients experienced excellent long-term pain relief, with a median pain score of 2 (range, 0–3 on a 0–10 scale) at a median follow-up of 66 months. Exocrine insufficiency improved in 10 patients, assessed by improvement of the fecal elastase level and cessation or reduction of pancreatic enzyme supplementation. However, endocrine insufficiency worsened in 4 out of 7 patients who required insulin supplementation to control their diabetes—not unexpected, given removal of the HOP and pre-existing pancreatic insufficiency.

The biliary reconstruction was carried out on the same loop of the PJ in 10 patients. Two patients developed bile leaks, possibly related to kinked biliary anastomoses. This prompted us to change the reconstruction technique in four patients by creating a double Roux-en-Y to reconstruct the PJ and the HJ. A fifth patient successfully underwent a novel reconstruction utilizing a gastric interposition graft created from the greater curvature of the stomach, without further incidence of bile leakage.

In conclusion, in this case series of 15 patients with CP, we show that PD combined with a modified Puestow procedure is feasible and safe, with excellent short-term and long-term pain relief, improved exocrine function, and better quality of life. It should be considered for patients with CP with calcifications involving the HOP and with strictures and stones along the MPD, but without involvement of the peripancreatic vasculature.
